# Temporal relationships between latent symptoms in psychosis: a longitudinal experience sampling methodology study

**DOI:** 10.1192/j.eurpsy.2023.308

**Published:** 2023-07-19

**Authors:** G. Gillett, D. Joyce, C. Karr, M. de Vos, D.-J. Dijk, N. Jacobson, J. MacCabe, N. Meyer

**Affiliations:** 1 Institute of Psychiatry, Psychology and Neuroscience, King’s College London, London; 2 Department of Psychiatry, University of Oxford, Oxford, United Kingdom; 3 Audacious Technologies, Chicago, United States; 4 Institute of Biomedical Engineering, University of Oxford, Oxford; 5 Sleep Research Centre, University of Surrey, Surrey, United Kingdom; 6 Center for Technology and Behavioral Health, Geisel School of Medicine, Dartmouth College, Lebanon, United States

## Abstract

**Introduction:**

A variety of dimensions of psychopathology are observed in psychosis. However, the validation of clinical assessment scales, and their latent variable structure, is often derived from cross-sectional rather than longitudinal data, limiting our understanding of how variables interact and reinforce one another.

**Objectives:**

Using experience sampling methodology (ESM) and analytic approaches optimised for longitudinal data, we assess potential latent variables of commonly-reported symptoms in psychosis, and explore the temporal relationship between them.

**Methods:**

N=36 participants with a diagnosis of schizophrenia or schizoaffective disorder provided data for up to one year, as part of the Sleepsight study. Using a smartphone app, participants self-reported clinical symptoms once daily for a mean duration of 323 days (SD: 88), with a response rate of 69%. Symptoms were rated using seven-point Likert scale items. Items included symptoms traditionally implicated in psychosis (feeling “cheerful”, “anxious”, “relaxed”, “irritable”, “sad”, “in control”, “stressed”, “suspicious”, “trouble concentrating”, “preoccupied by thoughts”, “others dislike me”, “confused”, “others influence my thoughts” and “unusual sights and sounds”). We used a sparse PCA (SPCA) model to identify latent variables in the longitudinal data. SPCA has previously been applied to longitudinal ESM data, and was developed to achieve a compromise between the explained variance and the interpretability of the principal components. We then used a multistage exploratory and confirmatory differential time-varying effect model (DTVEM) to explore the temporal relationship between the latent variables. DTVEM generates a standardised β coefficient reflecting the strength of relationship between variables across multiple time lags. Only significant lags (p<0.05) are reported here.

**Results:**

The SPCA analysis identified five latent variables, explaining 61.4% of the total variance. Tentative interpretation of the SPCA loadings suggested these latent variables corresponded to i) cognitive symptoms, ii) feeling in-control, iii) thought interference and perceptual disturbance, iv) irritability and stress and v) paranoia. Time lag analysis revealed an effect of feeling in-control on subsequent cognitive symptoms (β=-0.19), and of cognitive symptoms on subsequent thought interference and perceptual disturbance (β=0.14). Irritability and stress was also associated with subsequent cognitive symptoms (β=0.09).

**Conclusions:**

Using longitudinal data, we employ novel methodology to identify potential latent symptoms among commonly reported symptoms in psychosis. We identify five latent symptoms, and elucidate important temporal relationships between them. These findings may inform our understanding of the psychopathology of psychosis, potentially offering data-driven simplification of clinical assessment and novel insights for future research.

**Disclosure of Interest:**

None Declared

